# Selective DNA Gyrase Inhibitors: Multi-Target in Silico Profiling with 3D-Pharmacophores

**DOI:** 10.3390/ph14080789

**Published:** 2021-08-10

**Authors:** Tihomir Tomašič, Asta Zubrienė, Žiga Skok, Riccardo Martini, Stane Pajk, Izidor Sosič, Janez Ilaš, Daumantas Matulis, Sharon D. Bryant

**Affiliations:** 1 Faculty of Pharmacy, University of Ljubljana, Aškerčeva 7, 1000 Ljubljana, Slovenia; ziga.skok@ffa.uni-lj.si (Ž.S.); stane.pajk@ffa.uni-lj.si (S.P.); izidor.sosic@ffa.uni-lj.si (I.S.); janez.ilas@ffa.uni-lj.si (J.I.); 2 Department of Biothermodynamics and Drug Design, Institute of Biotechnology, Vilnius University, Saulėtekio 7, LT-10257 Vilnius, Lithuania; astzu@ibt.lt (A.Z.); matulis@ibt.lt (D.M.); 3 Inte:Ligand Softwareentwicklungs- und Consulting GmbH, Mariahilferstrasse 74B, 1070 Vienna, Austria; riccardo.martini@discngine.com (R.M.); bryant@inteligand.com (S.D.B.); 4 Discngine S.A.S., 79 Avenue Ledru Rollin, 75012 Paris, France

**Keywords:** antibacterial, ATP-competitive, DNA gyrase, Hsp90, pharmacophore model, topoisomerase II, target activity profiling

## Abstract

DNA gyrase is an important target for the development of novel antibiotics. Although ATP-competitive DNA gyrase (GyrB) inhibitors are a well-studied class of antibacterial agents, there is currently no representative used in therapy, largely due to unwanted off-target activities. Selectivity of GyrB inhibitors against closely related human ATP-binding enzymes should be evaluated early in development to avoid off-target binding to homologous binding domains. To address this challenge, we developed selective 3D-pharmacophore models for GyrB, human topoisomerase IIα (TopoII), and the Hsp90 N-terminal domain (NTD) to be used in in silico activity profiling paradigms to identify molecules selective for GyrB over TopoII and Hsp90, as starting points for hit expansion and lead optimization. The models were used to profile highly active GyrB, TopoII, and Hsp90 inhibitors. Selected compounds were tested in in vitro assays. GyrB inhibitors **1** and **2** were inactive against TopoII and Hsp90, while **3** and **4**, potent Hsp90 inhibitors, displayed no inhibition of GyrB and TopoII, and TopoII inhibitors **5** and **6** were inactive at GyrB and Hsp90. The results provide a proof of concept for the use of target activity profiling methods to identify selective starting points for hit and lead identification.

## 1. Introduction

The increasing emergence of pathogenic bacteria resistant to antibacterial drugs is a serious threat to global health and represents the continuous need for the development of novel antibacterial agents. In 2017, the World Health Organization published a list of priority pathogens, for which new antibacterial agents are urgently needed as the currently used therapy is becoming inefficient. Among these, resistant *Pseudomonas aeruginosa*, *Acinetobacter baumannii*, *Staphylococcus aureus*, *Enterococcus faecium*, and *Enterobacteriaceae* are of particular concern [1].

**Figure 1:**
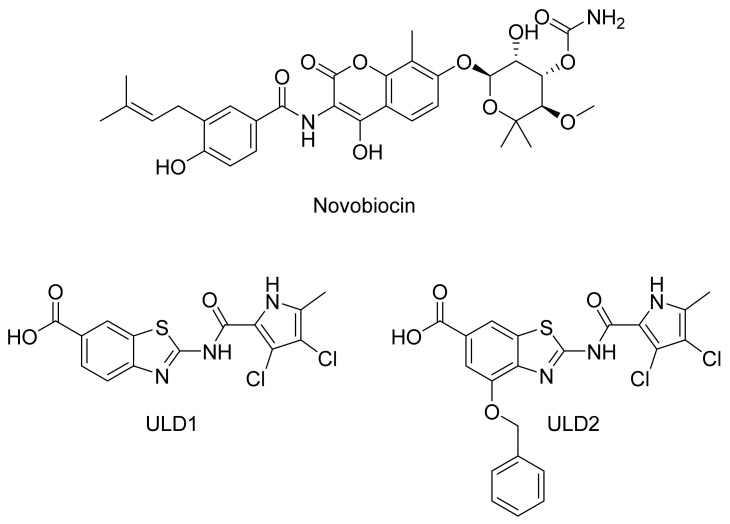
Structures of novobiocin and representative dual GyrB/ParE inhibitors ULD1 and ULD2. The ULD compounds displayed limited resistance development compared to novobiocin.

Numerous studies towards the development of new antibacterial agents have involved the design of novel ATP-competitive inhibitors of DNA gyrase [2,3,4,5,6,7,8,9,10]. DNA gyrase is an attractive target for antibacterial drug discovery because it plays an important role in the modulation of DNA topology during replication and is vital for the survival of the bacterial cell [10]. It is a heterotetramer that is composed of two catalytic GyrA subunits and two GyrB subunits with ATPase activity. While GyrA subunits are targets of the fluoroquinolone class of antibiotics, there is no representative GyrB inhibitor in the clinic. The only approved drug from the latter class used therapeutically in the past was novobiocin (Figure 1), which was withdrawn from the market because of its side effects and resistance development [9].

Targeting the ATP binding site of GyrB is a viable strategy toward the design of dual targeting inhibitors as they can bind also to the structurally similar ATP binding site of topoisomerase IV (ParE subunit). Such dual GyrB and ParE inhibitors have a prolonged onset of target-based resistance compared to compounds inhibiting only one bacterial target [11]. In a recent study, we have shown that compounds ULD1 and ULD2 (Figure 1) displayed balanced dual GyrB/ParE inhibition and retained activity against mutant strains comprising mutations in the target binding sites [12,13].

Selective toxicity is an important characteristic for the development of safe antibacterial drugs. As DNA gyrase is an ATP-binding protein, the design of inhibitors must address selectivity over other ATP-binding proteins in human cells. In many cases, although there is a low primary sequence homology between two proteins, they can share a higher similarity in the ATP-binding domain, which makes selective binding difficult to achieve [14]. For example, novobiocin, the only GyrB inhibitor ever used in the clinic, has been found to inhibit human TopoII [15] and weakly bind the allosteric binding site at the Hsp90 C-terminal domain (CTD) [16,17]. As TopoII and Hsp90 play important roles in the human cell, their inhibition is undesired for an antibacterial drug.

Human TopoII is a validated target in anticancer drug discovery and several drugs targeting this enzyme are used in therapy. However, there is no ATP-competitive TopoII inhibitor used as a drug for cancer treatment [18]. Hsp90 is a molecular chaperone with over 300 client proteins that act in the cell cycle and signaling processes [19]. Disruption of Hsp90 chaperone activity by inhibitors induces simultaneous proteasomal degradation of many deregulated oncoproteins that are critical for all fundamental hallmarks of cancers. Unlike targeting these individual oncoproteins, inhibition of Hsp90 will result in the degradation of more than 30 cancer targets simultaneously [20]. Several ATP-competitive Hsp90 inhibitors have entered clinical trials for various types of cancer. However, most of these trials were terminated due to toxicity or lack of efficacy [21]. All of these Hsp90 inhibitors bind to the ATP-binding site at the NTD and not the allosteric site at the Hsp90 CTD that was identified for novobiocin.

**Figure 2:**
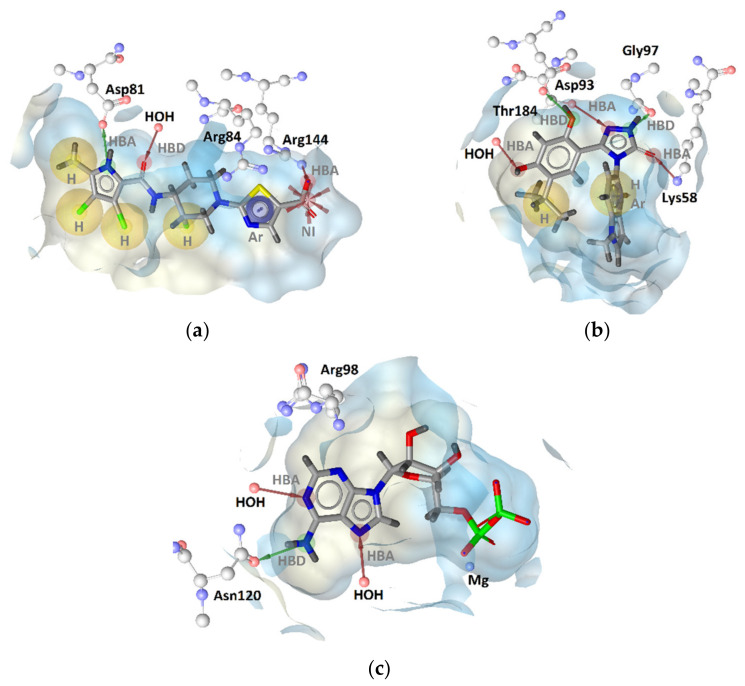
Comparison of crystal structures of (**a**) GyrB (PDB entry: 3TTZ) in complex with pyrrolamide-based inhibitor, (**b**) Hsp90 (PDB entry: 3TUH) in complex with inhibitor ganetespib, and (**c**) TopoII (PDB entry: 4R1F) in complex with ADP. The interaction features were derived using LigandScout 4.4 [29]. Hydrophobic features (H) are shown as yellow spheres, negative ionizable (NI) as a red star, aromatic ring (Ar) as a blue disc, hydrogen-bond donors (HBD) as green arrows, and hydrogen-bond acceptors (HBA) as red arrows.

Furthermore, it has recently been shown that it is possible to convert GyrB inhibitors into Hsp90 [22,23,24,25] or TopoII [26] inhibitors by the introduction of small structural changes in the ligands. However, selectivity against TopoII and Hsp90 can be achieved by exploiting important differences between these enzymes involving amino acid residues in the ATP-binding sites (Figure 2). While the hydrogen-bond (H-bond) donor and acceptor interactions with Asp or Asn and a structured water molecule and the presence of a magnesium ion (Figure 2c) in the binding site are important for substrate recognition, most of the GyrB inhibitors form strong interactions (H-bonds, cation-π stacking, salt bridge) with Arg144 (*S. aureus* GyrB numbering, Figure 2a), which is absent in the ATP-binding site of Hsp90 and TopoII [9]. Moreover, the Hsp90 NTD ATP-binding site is constricted by Lys58 and delineated by Asp102 with a negatively charged side chain (Figure 2b) that may prevent binding of a carboxylic acid moiety present in some GyrB inhibitors (Figure 1 and Figure 2a). Similarly, the TopoII ATP-binding site is constricted by the Arg98 side chain, which does not allow the binding of compounds to extend in its direction (Figure 2c). Moreover, selectivity profiles against human protein kinases are only rarely reported for GyrB inhibitors; however, in a couple of cases, these profiles were good indicating a potential for achieving selectivity [27,28].

Computer-aided drug design has become a state-of-the-art method in the discovery, rational design, and optimization of bioactive compounds. In particular, the concept of in silico target activity profiling for data mining and ligand selectivity profiling has been established for repurposing drugs, examining off-target liabilities, and identification of targets from phenotypic screening [30]. In addition, 3D-pharmacophores are highly useful and efficient tools for data mining (hit finding) and medicinal chemistry decision support in drug discovery research [31,32]. Furthermore, they have been reported to be highly efficient and accurate virtual screening (VS) filters when compared to docking and shape-based approaches [33]. In relevant studies, cyclothialidines were utilized to identify GyrB subunit inhibitors using 3D-pharmacophore, docking, and VS approaches [34]. Similarly, successful identification of Hsp90 N-terminal and C-terminal domain inhibitors was accomplished using 3D-pharmacophores and VS methodologies [35,36].

However, so far, the strategic use of 3D-pharmacophore models for multi-target activity profiling to identify GyrB inhibitors with selectivity over similar human ATP-binding domains, such as TopoII and Hsp90, has not been reported. This study involves the unique development of selective 3D-pharmacophore models for GyrB inhibition as a primary target and human Hsp90 and TopoII inhibition as off-targets to be used for multi-target in silico profiling of chemical structures. As inhibition of human TopoII or Hsp90 by a bacterial DNA gyrase inhibitor poses a severe risk for unwanted side effects due to their roles in modulation of DNA topology and protein homeostasis in healthy cells, the models would enable the high throughput identification and filtering of compounds with the potentially desired GyrB outcomes and not the undesired human Hsp90 and TopoII outcomes. This would be done strategically at an early stage of the molecular discovery process leading to the identification of higher quality chemical structure starting points.

To evaluate the usefulness of these models, we tested identified GyrB inhibitors for their inhibition of human TopoIIα in vitro and evaluated their binding to human Hsp90α. Furthermore, Hsp90 inhibitors were tested for inhibition of DNA gyrase and TopoII, and TopoII inhibitors were evaluated for their activity on Hsp90α and DNA gyrase.

## 2. Results and Discussion

### 2.1. Datasets for Model Design

For the identification of human proteins with similar binding sites to bacterial GyrB, we used ProBiS, a Web server for comparison of protein binding sites based on local structural alignments [37,38]. It enables the identification of proteins with similar binding sites by comparing the structure of a query protein with all protein structures in the Protein Data Bank. Therefore, similar ATP-binding sites can be detected even though proteins may have overall low sequence homology. In a previous study, the GyrB inhibitor novobiocin was shown to inhibit Hsp90 by binding to the CTD allosteric site (albeit weakly) and not the NTD ATP-binding site via the Bergerat ATP-binding fold identified by ProBiS [16,17]. In this study, a ProBiS query using *E. coli* GyrB (PDB ID: 4DUH) identified similar binding sites in Hsp90α, Hsp90β, and TopoIIα, which all belong to the GHKL (gyrase, Hsp90, histidine kinase, MutL) superfamily sharing the Bergerat ATP-binding fold [39]. Therefore, GyrB competitive inhibitors of the ATP-site could potentially interact in similar identified sites, and inhibition of human TopoII or Hsp90 by a bacterial DNA gyrase inhibitor poses a severe risk for unwanted damage to DNA or disruption of protein homeostasis in healthy cells.

For development and testing of 3D-pharmacophore models for prediction of inhibitors of human Hsp90 and TopoII, representing off-target effects, and bacterial DNA gyrase ATP site inhibition as the primary target, datasets of active compounds against GyrB (221 structures) and Hsp90 (649 structures) were retrieved from the ChEMBL database [40,41,42]. Active compounds for TopoII datasets (21 structures) were extracted manually from scientific publications. The cut-off for active compounds used for the creation of the models was set at K_d_ or IC_50_ of 100 nM or less in the case of GyrB and Hsp90, and 10 μM or less in the case of TopoII since no potent inhibitors are reported (IC_50_ < 100 nM). Designated inactive compounds had measured target affinities weaker than 100 μM.

In addition, for each active compound in the datasets, a set of 50 decoys was generated using the DUD-E (database of useful docking decoys) server [43]. The 3D-pharmacophore models were trained using their respective active and inactive datasets and tested using the respective active and decoy datasets. Receiver operating characteristic (ROC) curves were generated to compare the VS performance of the generated models. Models with steep curves showing a high rate of retrieval of true positive (TP), and low rate of false-positive (FP) compounds, an area under the curve (AUC) close to 1, and high enrichment factors (EF) were selected for the compound in silico activity profiling experiments involving the respective targets.

### 2.2. 3D-Pharmacophore Modelling

#### 2.2.1. DNA Gyrase B Inhibitors

ATP-competitive GyrB inhibitors belong to several structural classes, such as pyrrolamides, ethyl ureas, tricyclic inhibitors, arylaminopyrimidines, cyclothialidines, coumarins, and others, and display broad antibacterial activities against Gram-positive and Gram-negative strains [9]. Since many potent GyrB inhibitors belong to the pyrrolamide and ethyl urea structural classes, we focused structure-based pharmacophore modeling on GyrB complexes containing representative pyrrolamide- and ethyl urea-based inhibitors from X-ray crystallography studies. In addition, potent GyrB inhibitors with distinct structures in ChEMBL that have not been co-crystallized were used for ligand-based pharmacophore modeling to cover the chemical space of other reported active GyrB compounds.

##### GyrB Pyrrolamide Inhibitor Interaction Features

**Figure 3:**
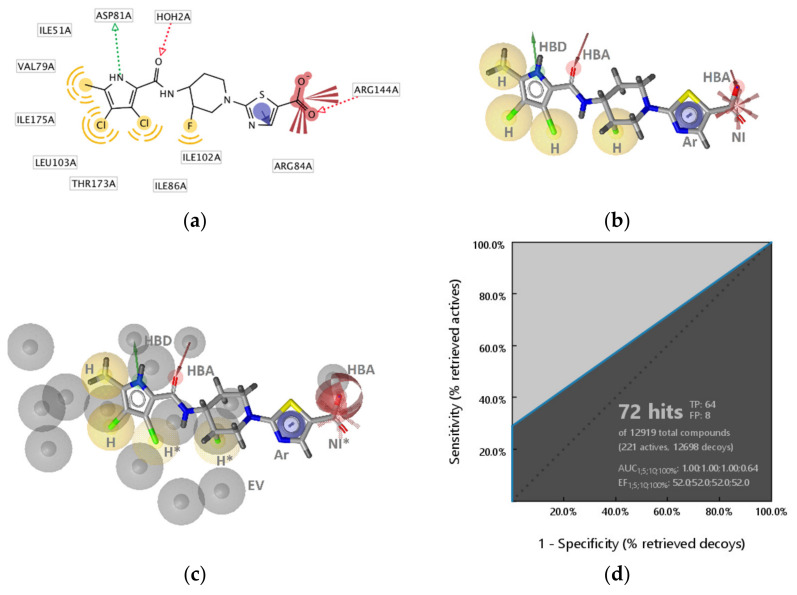
Structure-based binding interactions of a GyrB pyrrolamide inhibitor (PDB ID: 3TTZ) (**a**) 2D-depiction of interactions and the amino acid binding partners and (**b**) 3D-depiction of the SB-interaction model (binding site interactions displayed in Figure 2a); (**c**) **SB-GyrB-Model-1**: refined structure-based GyrB inhibitor pharmacophore model based on a pyrrolamide inhibitor. The pharmacophore features are: hydrophobic (H, yellow spheres), aromatic (Ar, blue disc), hydrogen bond donor (HBD, green arrow), hydrogen bond acceptor (HBA, red arrow or sphere), negative ionizable (NI, red star). Exclusion volumes (EV, grey). Optional pharmacophore features are marked with *; (**d**) ROC plot (curve shown in blue) generated from virtually screening 12,919 compounds (221 GyrB actives and 1,2698 generated decoys) with the **SB-GyrB-Model-1**. TP = true positives; FP = false positives; AUC = area under the curve; EF = enrichment factor.

The X-ray crystallography resolved structure of *S. aureus* GyrB in complex with a pyrrolamide-based inhibitor (PDB ID: 3TTZ) [44] was used for identifying key interactions between the ligand and the binding site in a direct approach using LigandScout 4.4 [45]. The interactions consisted of four hydrophobic features, an aromatic feature, two hydrogen bond acceptors (HBA), a hydrogen bond donor (HBD), and a negative ionizable feature consistent with observations reported in the X-ray analysis (Figure 2a and Figure 3a,b). Virtual screening of the GyrB structure-based (SB) model against the active and decoy datasets revealed a specific binding mode of this inhibitor, as the 3D-interaction model did not retrieve any other actives nor decoys from the dataset. To explore structure-activity relationships and if the model could retrieve more actives, the interaction features were modified (Figure 3c). The hydrophobic features on the fluorine atom of the piperidine ring, the chlorine atom at position 3 of the pyrrole moiety, and the negative ionizable feature of the carboxylate were marked as optional. Moreover, the directional HBA interaction feature associated with Arg144 (*S. aureus* numbering) was converted to a sphere. In the context of VS (hit finding), the optional features are not required to be matched for a molecule to be retrieved. However, if they are matched the pharmacophore fit score will be higher and the virtual hit will have a higher ranking. In addition, the tolerance of the directional aromatic feature was increased by 0.30 Å making the feature less restrictive though still required. When the aromatic feature was disabled, inactives and decoys were retrieved, indicating the importance of the position of the feature. In fact, the majority of GyrB inhibitors have an aromatic ring and likely form a cation-π interaction with Arg84 (*S. aureus* numbering). The refined **SB-GyrB-Model-1** (Figure 3c) performed very well, retrieving 30% (64 actives) of the true positives and 0.02% (8 decoys) false positives, an enrichment factor (EF) of 52.0 and AUC of 1 at 1, 5, 10% of screening the dataset of 12,919 compounds (Figure 3d). Among the 64 hits, 62 were pyrrolamides and 2 indazole inhibitors. Further analysis revealed that the directional hydrogen bond donor (HBD) associated with Asp81, the nearby hydrophobic feature, the hydrogen bond acceptor (HBA) associated with the structured water molecule, and the HBA associated with Arg144 are likely prerequisites for GyrB ATP binding site inhibition and are common in all of the 64 retrieved active GyrB inhibitors. Furthermore, the aromatic feature associated with Arg84 plays a role in alignment and distinguishing active from inactive molecules. When disabling the other three hydrophobics, associated with Ile86, Ile102, and Thr173, and the negative ionizable feature, the model retrieved more than 50% of the true actives indicating that those features are not required for activity but are likely important for selectivity or enhanced inhibition activities (Figure 3c).

##### GyrB Ethyl Urea Inhibitor Interaction Features

**Figure 4:**
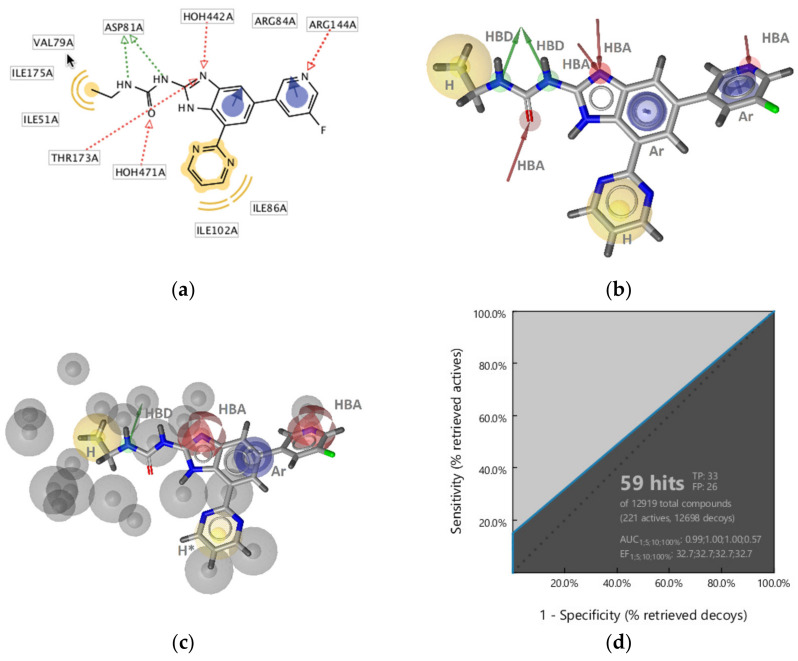
3D-Structure-based interactions derived from the X-ray structure of GyrB and an ethylurea inhibitor (PDB ID: 4P8O): (**a**) 2D-depiction with binding site interaction partners and (**b**) 3D-depiction of initial SB-pharmacophore model with the ligand; (**c**) The refined **SB-Gyr-Model-2**, (**d**) ROC plot (curve shown in blue) from virtually screening 12,919 compounds (221 GyrB actives and 12,698 generated decoys) with **SB-GyrB-Model-2**. TP = true positives; FP = false positives; AUC = area under the curve; EF = enrichment factor. Displayed pharmacophore features are: hydrophobic (H, yellow spheres), aromatic (Ar, blue disc), hydrogen bond donor (HBD, green arrow), and hydrogen bond acceptor (HBA, red arrow and red sphere). Optional pharmacophore features are marked with *.

Some interactions derived from the X-ray resolved crystal structure of *S. aureus* DNA gyrase in complex with an ethyl urea-based inhibitor (PDB ID: 4P8O) [46] were similar to those observed with the pyrrolamide inhibitor (Figure 3a and Figure 4a). Similar features consisted of two hydrophobic interactions involving Ile51, Val79, Ile86, and Ile102, as well as the HBD (Asp81), HBA (structured water molecule), aromatic (Arg84), and HBA (Arg144). In contrast, additional HBA interactions were observed with Thr173 and benzimidazole nitrogen as well as between a water molecule and the urea oxygen, and an aromatic interaction was captured between the benzimidazole aromatic and Arg84 (Figure 4a,b). **SB-GyrB-Model-1** had two hydrophobic features related to chlorine substituents that are not present in the GyrB-ethyl urea interaction model (Figure 3a). The GyrB-ethyl urea model identified 6% of the active compounds (13 true positives) and no false positives. All 13 hits contained the urea scaffold and 12 of the 13 contained a benzimidazole scaffold. To explore the role of the position of the aromatic interactions and attempt to cover more of the active space the model was modified. The aromatic feature interacting with Arg144 was removed. The 3D-geometry was similar to the aromatic in **SB-GyrB-Model-1**, though the interaction partner was Arg84. The HBA directional features were converted to spheres (Figure 4c). The tolerances of aromatic, the hydrophobic features associated with the ethyl group and pyrimidine ring as well as the HBAs on the N-3 of the benzimidazole were increased and the latter was marked optional. The HBD feature of the ethylamine of the ethyl urea was retained, while the other HBD and HBA features of the ethyl urea were removed. The optimized model retrieved 14.9% of the actives (33 true positives) and 0.20% (26 decoys), resulting in better coverage of the active space albeit, the overall hit rates were not as good as those achieved with **SB-GyrB-Model-1** as shown in Figure 4d. However, the two models identified different scaffolds. **SB-GyrB-Model-1** did not retrieve any of the GyrB inhibitors containing ethyl urea scaffolds because of the required hydrophobic feature that would be in a similar space as the urea carbonyl oxygen (HBA). **SB-GyrB-Model-2** identified (33/40) of the ethyl urea structural class of compounds in the actives dataset and none of the GyrB inhibitors that **SB-GyrB-Model-1** retrieved. The results suggest that the urea oxygen or an HBA in that position would not be a prerequisite for inhibitors in the ATP binding site. 

##### GyrB Inhibitor Ligand-Based Pharmacophore Models

**Figure 5:**
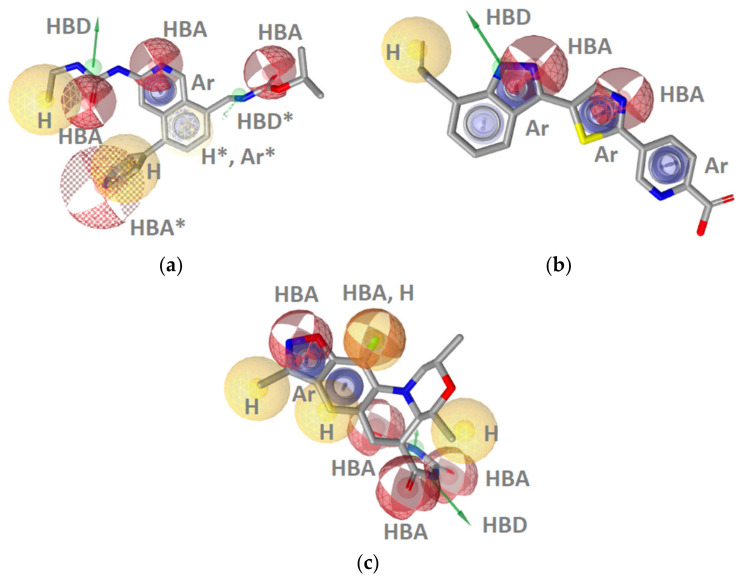
Three of the best performing GyrB inhibitor ligand-based models resulting from clustering a dataset of 221 very active GyrB inhibitors: (**a**) **LB-GyrB-Model-5m**; (**b**) **LB-GyrB-Model-4**; (**c**) **LB-GyrB-Model-6**. The pharmacophore features are as follows: hydrophobic (H, yellow spheres), aromatic (Ar, blue disc), hydrogen bond donor (HBD, green arrow), and hydrogen bond acceptor (HBA, red sphere). Optional pharmacophore features are marked with *.

As the SB models covered around 45% of the active space, we utilized ligand-based approaches to develop additional models to cover the unaddressed active space. The multiconformational dataset of 221 GyrB inhibitors (IC_50_ values below 100 nM) was clustered based on their 3D-pharmacophore features. A total of 16 ligand-based models were developed based on the clusters and tested against the active and inactive datasets. The three best performing GyrB inhibitor LB-models (**Models 5m, 4 & 6**) are shown in Figure 5. The ROC plots are included in the Supporting information (Figure S1). The models contained 7–13 features and performed well on the validation datasets. They had low false positive hit rates (0–0.008%) and high enrichment factors (EF = 57–58). Interestingly, all of the LB models derived using only ligand information contained the hydrophobic, HBD, HBA, and aromatic interaction feature patterns observed in the structure-based models that were derived using GyrB binding site information from X-ray crystal structures.

**LB-GyrB-Model-5m** had the fewest number of features (7) and retrieved the highest number of true positives (18%), compared to the other ligand-based models (Figure 5a). It retrieved only ethyl urea derivatives, though the derivatives retrieved contained other substructures, such as isoquinolines, benzothiazoles, thiazoles, pyrazoles, and pyrrolopyridines. **LB-GyrB-Model-4** retrieved 18% of the active GyrB inhibitors in the dataset, all containing indazole scaffolds that were not retrieved by the other models (Figure 5b). **LB-GyrB-Model-6** retrieved 2% of the actives containing only benzothiazole derivatives. Interestingly, the model lacked an HBD feature in the position of the hydrophobic, HBD, HBA pattern observed in the SB-models (Figure 5c). 

**Figure 6:**
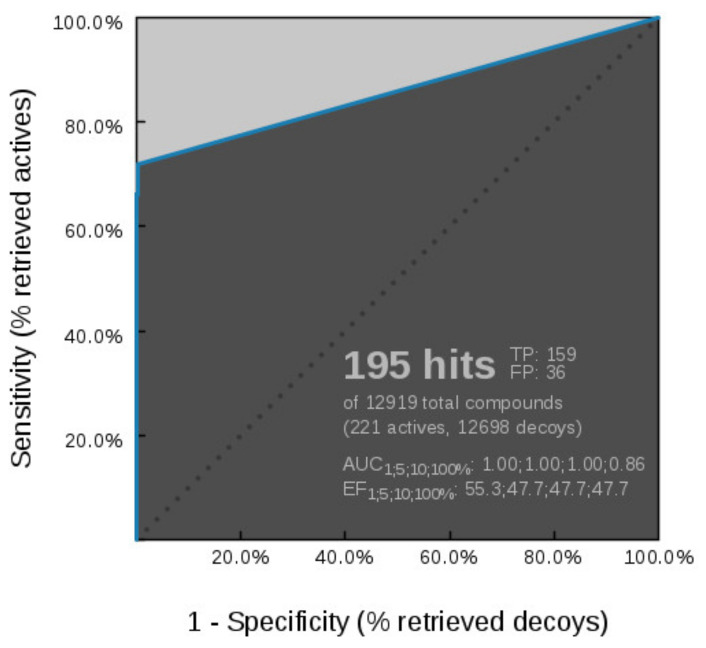
Receiver operating characteristic (ROC) curve resulting from parallel virtual screening of the GyrB active and decoy datasets with five GyrB inhibitor 3D-pharmacophore models. The parallel screening approach gives a high coverage of GyrB inhibitor active space while maintaining a low false-positive rate.

The five prioritized GyrB inhibitor pharmacophore models were screened in parallel against the GyrB actives and decoy sets. The resulting ROC curve is shown in Figure 6. **SB-GyrB-Model-1** and **LB-GyrB-Model-4** retrieved 2 identical ligands, which belong to the pyrrolamide class of inhibitors, while **SB-GyrB-Model-2** and **LB-GyrB-Model-5m** retrieved 20 identical ligands. The parallel VS strategy enables a high coverage of the GyrB active space (71.9%) while maintaining low false-positive rates (0.3%). Furthermore, the set of GyrB pharmacophore models was used to screen a library of 29 GyrB inhibitors with IC_50_ values ranging from 100 nM to 1 μM, and 9 compounds (31.0% hit rate) were recovered. The strategy provides a robust solution for mining large libraries of commercially available and/or in-house compound collections to identify GyrB inhibitors.

##### Virtual Screening of Hsp90 and TopoII Inhibitors with GyrB Pharmacophore Models

**Figure 7:**
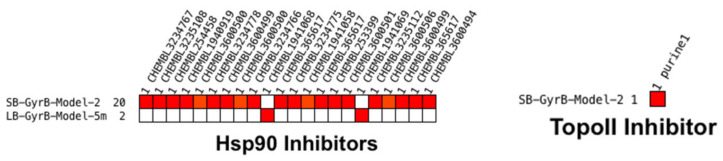
Heatmaps were obtained after screening 649 of the most potent Hsp90 inhibitors (K_d_ < 100 nM) and 21 TopoII inhibitors using 5 GyrB 3D-pharmacophore models. **SB-GyrB-Model-2** retrieved both Hsp90 and TopoII inhibitors, and **LB-GyrB-Model-5m** retrieved only 2 Hsp90 inhibitors. None of the other GyrB models retrieved compounds from the Hsp90 and TopoII datasets.

Virtual screening of the 649 Hsp90 inhibitors and 21 TopoII inhibitors with the 5 GyrB inhibitor pharmacophore models resulted in 1 TopoII and 20 Hsp90 inhibitor hits (Figure 7). **SB-GyrB-Model-2** retrieved one TopoII and 20 of the Hsp90 inhibitors, while **LB-GyrB-Model-5m** retrieved only 2 Hsp90 inhibitors. None of the other GyrB models retrieved hits from those datasets. **SB-GyrB-Model-2** contains chemical features that are common to all three target sites. To investigate the role of these features in selectivity the optional hydrophobic feature was marked required and the Hsp90 and TopoII datasets were screened again. The modified model retrieved the TopoII molecule and 16 of the Hsp90 inhibitors. The three hydrophobic features near the hydrophobic, HBD, HBA pattern observed in **SB-GyrB-Model-1** may play an important role in selective GyrB inhibition over Hsp90 and TopoII inhibitors. 

#### 2.2.2. Heat Shock Protein 90 Alpha (Hsp90α) Inhibitors

Selectivity of GyrB inhibitors against human Hsp90 is seldom reported, even though they share a similarity in the ATP-binding fold [39] and that novobiocin was shown to bind Hsp90 CTD allosteric binding site [16,17]. We believe it is important to assess GyrB inhibitors for Hsp90 inhibition since non-selective compounds could exert unwanted side effects due to cytotoxic behavior by affecting protein homeostasis through binding to Hsp90.

Furthermore, in silico profiling of compounds targeting GyrB with Hsp90 models will be useful to prioritize selective compounds at an early stage. To do this structure- and ligand-based 3D-pharmacophores were developed to predict Hsp90 ATP binding site inhibitors. In a previous study, we reported the development of pharmacophores for Hsp90 C-terminal domain inhibition [36]. X-ray structures of Hsp90 in complex with resorcinol and dihydropyridopyrimidinone classes of inhibitors were identified for structure-based pharmacophore modeling. In addition, a set of ligand-based pharmacophore models was created based on the dataset of Hsp90 inhibitors with K_d_ or IC_50_ values below 100 nM.

##### Hsp90 Resorcinol Interaction Features

**Figure 8:**
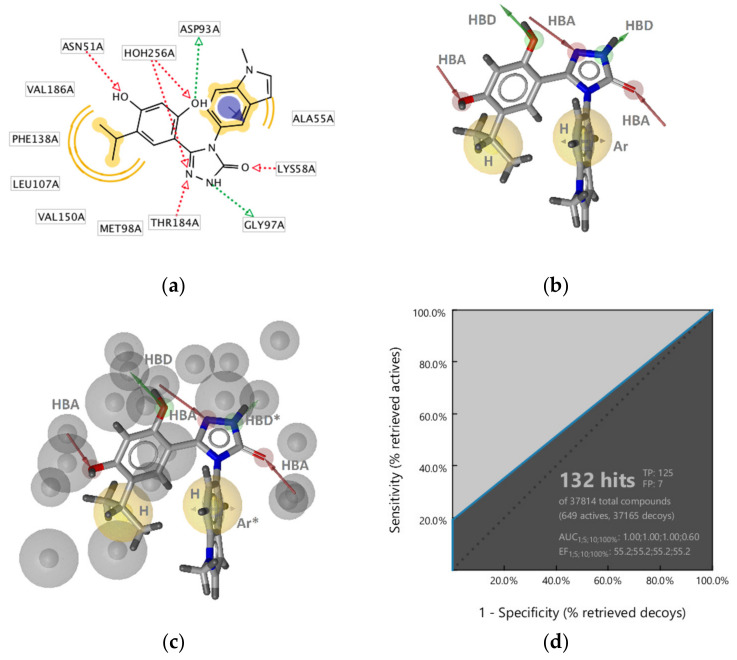
3D-structure-based interactions of the inhibitor ganetespib derived from an Hsp90 X-ray structure (PDB ID: 3TUH). (**a**) 2D-depiction with binding site interaction partners and (**b**) 3D-depiction of the directly derived SB-pharmacophore model; (**c**) The refined SB-Hsp90-Model-1, (**d**) Receiver operating characteristic (ROC) plot (curve shown in blue) derived from virtually screening 37,814 compounds (649 Hsp90 actives and 37,165 decoys) with SB-Hsp90-Model-1. TP = true positives; FP = false positives; AUC = area under the curve; EF = enrichment factor. Displayed pharmacophore features are: hydrophobic (H, yellow spheres), aromatic (Ar, blue disc), hydrogen bond donor (HBD, green arrow), and hydrogen bond acceptor (HBA, red arrow). Optional pharmacophore features are marked with *.

Interaction features of the resorcinol inhibitor ganetespib with the Hsp90α ATP-binding site were derived using the X-ray crystal structure (PDB code 3TUH). The model consisted of five HBAs, two HBDs, one aromatic, and two hydrophobic features (Figure 8a,b). The hydrophobic, HBD (Asp93), HBA (structured water), aromatic, and HBA, both associated with a positively charged amino acid (Lys58) were analogous to interaction features observed in the GyrB ATP site inhibitors (Figure 3a, Figure 4a and Figure 8a). However, the HBA (Asn51) between the hydrophobic feature and the HBD was not observed in the GyrB SB-models nor was the additional HBD with Gly backbone carbonyl.

The model retrieved 9 of the 649 Hsp90 inhibitors. To decrease the specificity of the model, the HBD on the triazole and aromatic ring were marked optional (Figure 8c). The refined **SB-Hsp90-Model-1** retrieved 19% (125) of active Hsp90 compounds and only 0.02% of the decoys resulting in AUC and EF values of 1.0 and 55.2, respectively (Figure 8d) indicating that the HBD (green) interaction between the triazole NH and the backbone carbonyl oxygen of Gly97 was a feature specific to certain derivatives and not required for activity at Hsp90.

##### Hsp90 Dihydropyridopyrimidinone Interaction Features

**Figure 9:**
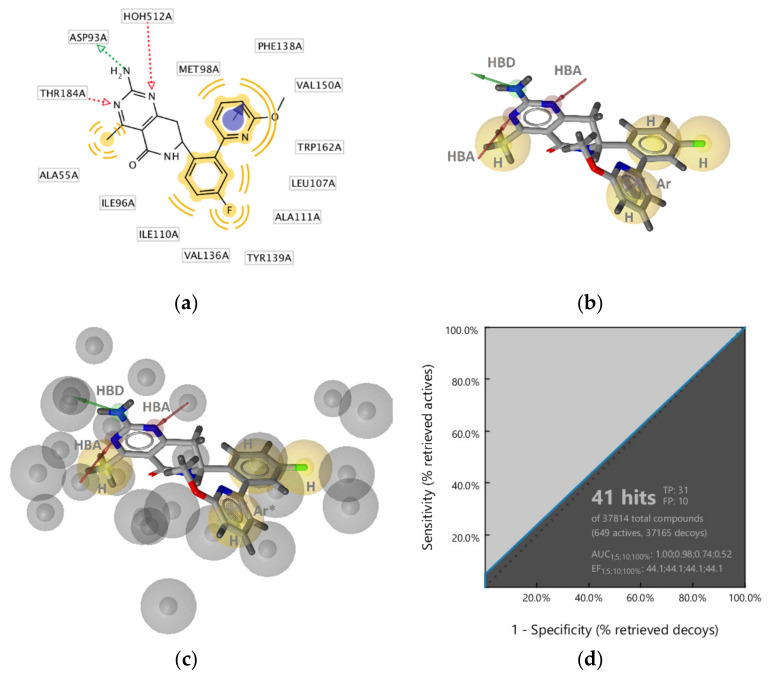
3D-structure-based interactions derived from a dihydropyridopyrimidinone Hsp90 inhibitor (PDB ID: 4U93): (**a**) 2D-depiction with binding site interaction partners and (**b**) 3D-depiction the SB-pharmacophore model with the ligand; (**c**) The refined SB-Hsp90-Model-2, (**d**) Receiving operating characteristic (ROC) (curve shown in blue) from virtually screening 37,814 compounds (649 Hsp90 actives and 37,165 generated decoys) with the refined SB-Hsp90-Model-2. TP = true positives; FP = false positives; AUC = area under the curve; EF = enrichment factor. Displayed pharmacophore features are: hydrophobic (H, yellow spheres), aromatic ring (Ar, blue disc), hydrogen bond donor (HBD, green arrow), and hydrogen bond acceptor (HBA, red arrow). Optional pharmacophore features are marked with *.

Interaction features of a dihydropyridopyrimidinone inhibitor with the Hsp90α ATP-binding site (PDB ID: 4U93 [47]) consisted of an HBD, two HBAs, one aromatic, and four hydrophobic features (Figure 9a,b). A similar interaction pattern to features in **SB-Hsp90-Model-1** involving the hydrophobic (Ala55), HBD (Asp93), HBA (structured-water), and aromatic-hydrophobic (Phe138, Tyr139, Trp162) features was noted (Figure 8a and Figure 9a). In contrast, the HBA (Thr184), and aromatic-hydrophobic features had different interaction partners compared to ganetespib (Figure 8a and Figure 9a) while the additional hydrophobic features associated with dihydropyridopyrimidinone inhibitor were unique to this inhibitor. To identify more true active hits, from the Hsp90 actives set, the restrictive aromatic feature was marked optional (Figure 9c). The model retrieved 5% (31) active compounds and 0.03% of the decoys and an enrichment factor of 44.1 (Figure 9d). Though the model retrieved fewer true positives than **SB-Hsp90-Model-1**, all of the actives retrieved contained the dihydropyridopyrimidinone substructure. Furthermore, the two models did not retrieve the same compounds, indicating unique binding features associated with these derivatives, which may be important for selectivity considerations when designing compounds for or against this target.

##### Hsp90 Ligand-Based (LB) Pharmacophore Models

The two Hsp90 ATP binding site SB models retrieved around 24% of the Hsp90 inhibitors in the test set. To further explore the active space, the 649 Hsp90 actives were clustered based on their pharmacophore radial distribution function (RDF)-code similarity, and LB-pharmacophore models were generated using selected clusters of compounds. Altogether, a set of 30 ligand-based pharmacophore models was generated for Hsp90 inhibitors.

Four models resulting from the most populated clusters and those with complex molecules, such as geldanamycin, its analogs, argenteoside A and B, and other natural compounds had low predictive power due to high false-positive retrieval rates. The poor selectivity was largely due to fewer overall common features and the less restrictive exclusion volume space that was added around the shape of the larger sets of diverse molecules. To address these issues, the largely populated clusters were divided into smaller clusters based on pharmacophore alignment scores as a similarity measure. The resulting models, derived from smaller sets of molecules, selectively retrieved most of the Hsp90 inhibitors from the dataset with almost no decoys giving more robust models for Hsp90 ATP site target selectivity screening purposes.

**Figure 10:**
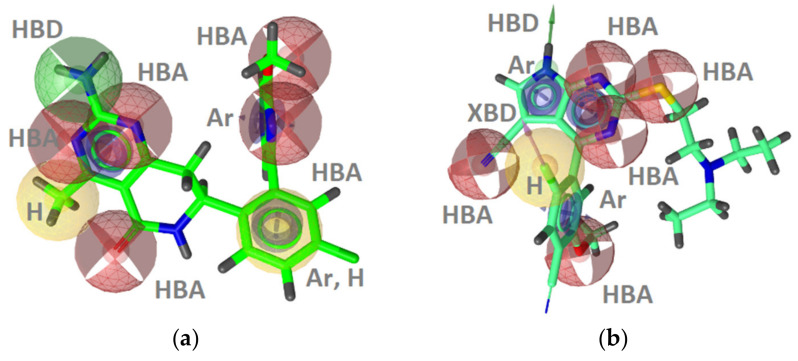

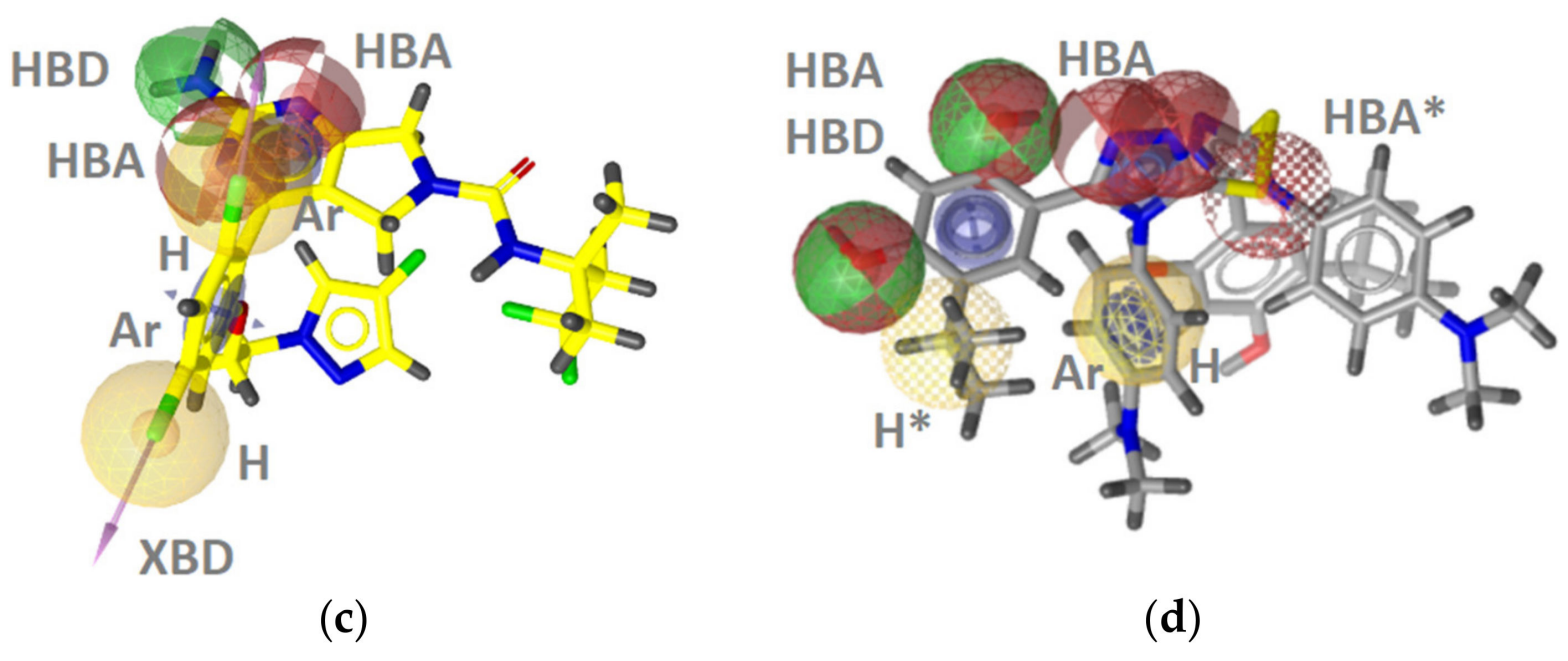
(**a**) **LB-Hsp90-Model-3**, (**b**) **LB-Hsp90-Model-4**, (**c**) **LB-Hsp90-Model-5**, and (**d**) **LB-Hsp90-Model-6** Hsp90 inhibitor ligand-based pharmacophore models. The pharmacophore features are as follows: hydrophobic (H, yellow spheres), aromatic (Ar, blue disc), hydrogen bond donor (HBD, green arrow, or green sphere), hydrogen bond acceptor (HBA, red sphere), and halogen bond (XBD, pink arrow). Optional pharmacophore features are marked with *.

Four of the thirteen best performing LB-pharmacophore models for Hsp90 ATP binding site inhibitors are shown in Figure 10 and the corresponding ROC plots are included in the supporting information (Figure S2). All of the LB-models contained the hydrophobic, HBA, HBD, and HBA, patterns observed in **SB-Hsp90-Models-1** and **-2** (Figure 8, Figure 9, Figure 10) even though SB information was not used to create the LB-models. HBD and HBA features that interact with Asp93 and structured water molecules in the Hsp90 ATP binding site are important as they mimic the binding mode of the adenine ring of ATP. Corresponding structured-water molecules and amino acids Asp81 and Asn120 are present in GyrB and TopoII ATP binding sites (Figure 2). In addition, they all contained aromatic interaction features in similar locations as noted in the SB-models emphasizing the importance of π-π stacking or cation-π interactions in the binding sites with either Phe138 or Lys58 (Hsp90), Arg84 (GyrB), or Arg98 (TopoII).

**Figure 11:**
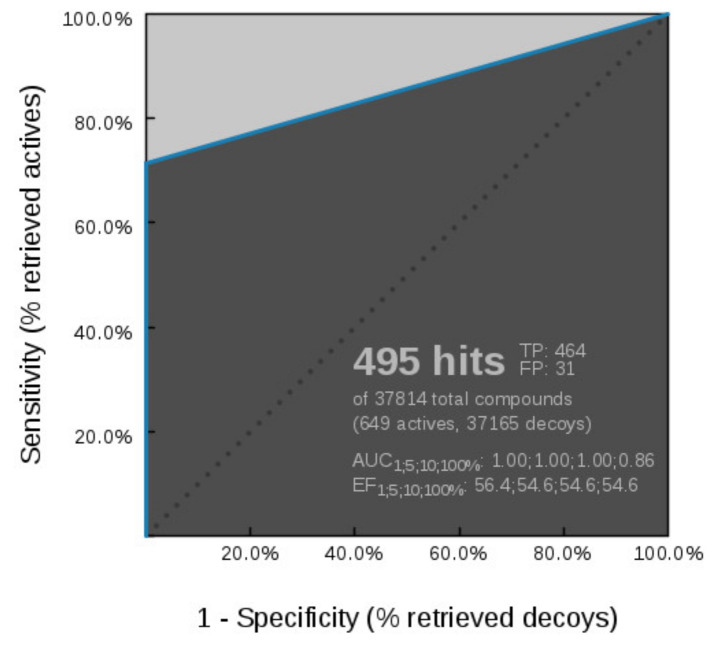
Receiver operating characteristic (ROC) curve resulting from parallel virtual screening of the Hsp90 active and decoy datasets with 13 Hsp90 inhibitor 3D-pharmacophore models. The parallel screening approach gives a high coverage of Hsp90 inhibitor active space while maintaining an acceptable false-positive rate.

All of the prioritized Hsp90 SB- and LB-pharmacophore models were screened in parallel against Hsp90 actives and decoys sets. The resulting ROC curve from screening is shown in Figure 11. Screening with 13 LB- and 2 SB-models covered 71.5% of the Hsp90 active space with 0.08% of false positives, resulting in a set of models suitable for selective target activity profiling for Hsp90 ATP site inhibition. **SB-Hsp90-Model-1** was able to retrieve 36 unique ligands (36/125), while the majority of the remaining ligands overlapped with hits found by **LB-Hsp90-Model-6**. **SB-Hsp90-Model-2** identified 9 unique inhibitors (9/30), while other hits overlapped mostly with hits found by **LB-Hsp90-Model-3**. Furthermore, screening 512 Hsp90 inhibitors with K_d_ or IC_50_ values between 100 nM and 1 μM with the Hsp90 pharmacophore model set identified 244 hits (47.7% hit rate), demonstrating that hits with weaker activities are also successfully identified in compound libraries.

##### Virtual Screening of GyrB and TopoII Inhibitors with Hsp90 Pharmacophore Models

Virtual screening of the 221 GyrB inhibitors and 21 TopoII inhibitors with the Hsp90 pharmacophore models did not identify any GyrB or TopoII inhibitor hits. GyrB inhibitors generally could not match the required HBA feature associated with Asn51 or Thr184 in Hsp90 within with the noted hydrophobic, HBA, HBD, HBA binding pattern (Figure 2, Figure 8 and Figure 9). Instead, GyrB inhibitors display a hydrophobic, HBD, HBA binding pattern (Figure 2, Figure 3, Figure 4). The HBA between the hydrophobic and HBD is an important feature for Hsp90 ATP binding site selectivity.

#### 2.2.3. Human Topoisomerase IIα Inhibitors

Like Hsp90, inhibition of human TopoII by a GyrB antibacterial inhibitor could result in unwanted cytotoxicity and undesired side effects. Though the binding sites share similarities, evaluation of selectivity in vitro is seldom reported [48]. However, differences in ATP-binding sites of GyrB and TopoII can be exploited to achieve selectivity. To do this using in silico approaches, 3D-pharmacophore models were developed using the most potent inhibitors available. X-ray structures with TopoII inhibitors have not been reported so far and therefore SB-models were not developed in this study.

##### TopoII Inhibitor Ligand-Based Pharmacophore Models

**Figure 12:**
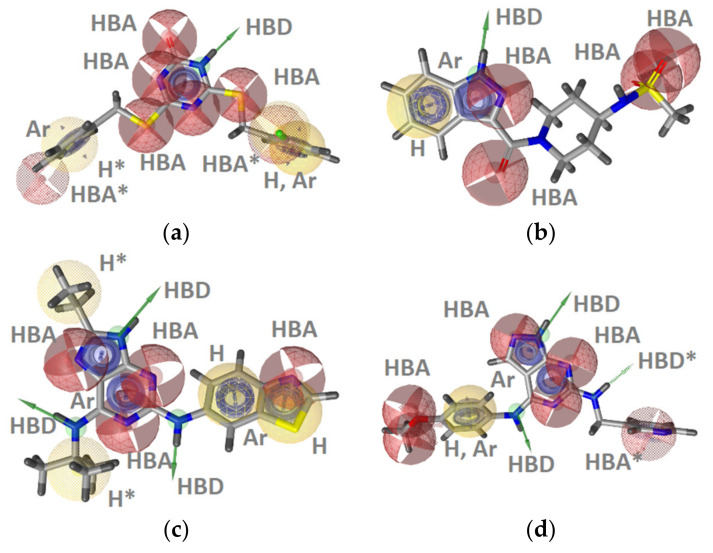

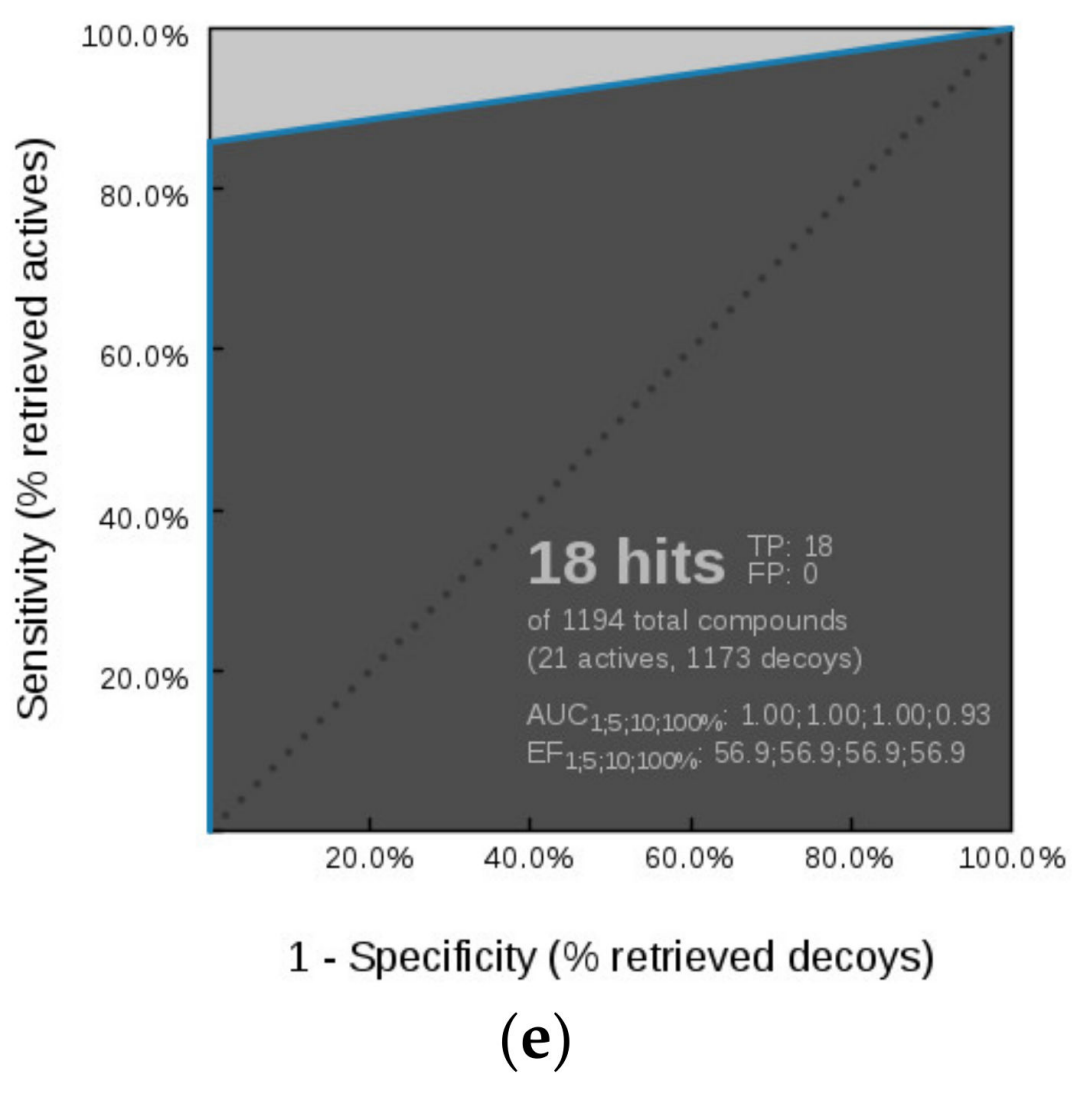
TopoII inhibitor ligand-based pharmacophore models: (**a**) **LB-TopoII-Model-1**, (**b**) **LB-TopoII-Model-2**, (**c**) **LB-TopoII-Model-3**, and (**d**) **LB-TopoII-Model-4**. The pharmacophore features are as follows: hydrophobic (H, yellow spheres), aromatic (Ar, blue disc), hydrogen bond donor (HBD, green arrow), and hydrogen bond acceptor (HBA, red sphere). Optional pharmacophore features are marked with *. (**e**) Receiver operating characteristic (ROC) curve resulting from parallel virtual screening of the TopoII active and decoy datasets with the 4 TopoII inhibitor 3D-pharmacophore models.

**Figure 13:**
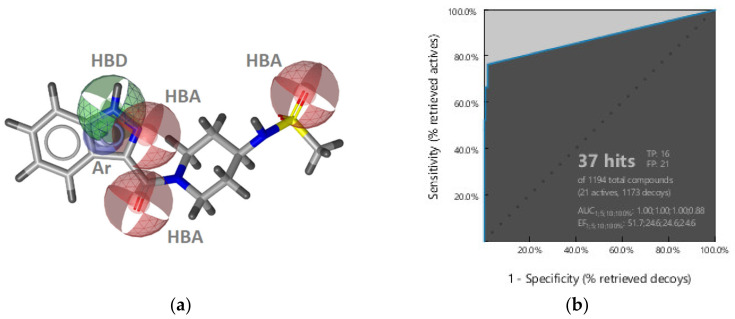
(**a**) **LB-TopoII-Model-5**, a consensus model derived from four selective TopoII inhibitor ligand-based pharmacophore models. The pharmacophore features include aromatic (blue disc), hydrogen bond donor (HBD, green arrow), and hydrogen bond acceptor (HBA, red sphere). (**b**) ROC plot (curve shown in blue) from virtually screening 1194 compounds (21 TopoII actives and 1173 decoys) with **LB-TopoII-Model-5**. TP = true positives; FP = false positives; AUC = area under the curve; EF = enrichment factor.

The TopoII actives dataset consisted of 21 inhibitors with IC_50_ values below 10 μM, for which binding to the ATP-binding site was confirmed by a relevant in vitro assay [18]. The inhibitors were clustered based on pharmacophore RDF-code similarity. Four ligand-based pharmacophore models were generated (Figure 12). Parallel screening of the test datasets resulted in 86% of the actives and none of the decoys (Figure 12e). The models had between 8–15 features making them highly specific (Figure S3) and suitable for the identification of molecules with similar scaffolds that would be selective for TopoII ATP binding sites. Though the TopoII actives data set was small compared to the GyrB and Hsp90 actives sets, the parallel screening approach gave a high coverage of the known TopoII inhibitor active space while identifying no false positives. The creation of a consensus model based on the four feature-rich LB models revealed an aromatic, HBD, and 3 HBAs in the same geometries (Figure 13). The HBD and HBA features close to an aromatic feature were analogous to feature patterns observed in the Hsp90 and GyrB pharmacophore models (Figure 2). In fact, the HBD and HBA features likely represent signature ATP binding site interactions with Asn120 and the structured water molecule, respectively, and form the same interactions as the adenine ring of ATP (Figure 2). Similarly, the HBA associated with the sulphoxide carbonyl group likely interacts with Arg98 as analogous features were observed in the inhibitor binding sites (Arg84 and Lys58) of GyrB and Hsp90 (Figure 2). The consensus feature TopoII inhibitor pharmacophore model (**LB-TopoII-Model-5**) was screened against the TopoII active and decoy datasets and retrieved 76.2% of the true actives and 1.8% of the false positives (Figure 13b). The consensus model would be useful for identifying GyrB compounds with risk for TopoII inhibition activity.

##### Virtual Screening of GyrB and Hsp90 Inhibitors with TopoII Pharmacophore Models

**Figure 14:**
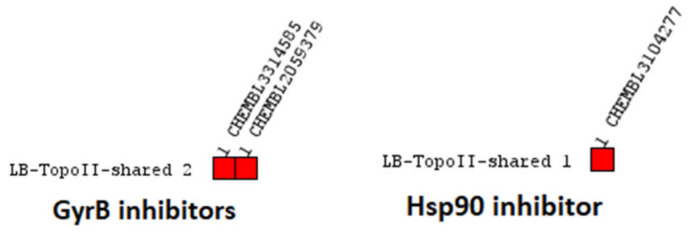
Heatmaps obtained after screening 221 of the most potent GyrB inhibitors (IC_50_ < 100 nM) and 649 Hsp90 inhibitors (K_d_ < 100 nM) using TopoII 3D-pharmacophore models. **LB-TopoII-Model-5**, a shared feature (consensus) model retrieved both GyrB and Hsp90 inhibitors.

Virtual screening of the 221 GyrB inhibitors and 649 Hsp90 inhibitors with the 5 TopoII inhibitor pharmacophore models resulted in the identification of 2 GyrB and 1 Hsp90 inhibitor hits (Figure 14). The hits were identified by the shared feature **LB-TopoII-Model-5**, while none of the feature-rich, highly specific LB models identified hits.

### 2.3. Biological Evaluation

**Figure 15:**
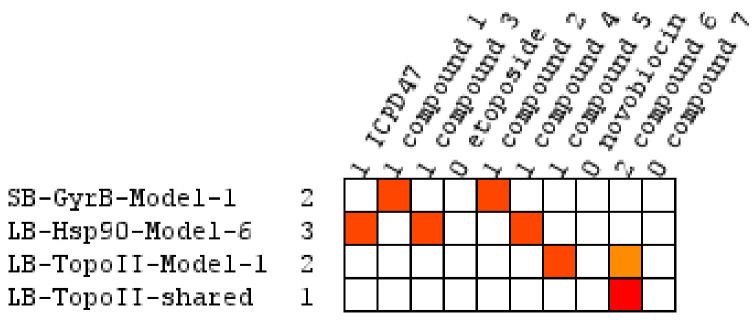
Virtual screening hits in the activity profiling of compounds **1**–**7** and positive controls ICDP47B, novobiocin, and etoposide using GyrB, Hsp90, and TopoII pharmacophore models.

Our in-house library of 257 pyrrolamide-based GyrB inhibitors was profiled with the prioritized sets of GyrB, Hsp90, and TopoII pharmacophore models, which were predicted to selectively inhibit GyrB. To confirm this result experimentally, *Escherichia coli* DNA gyrase inhibitors **1** and **2** (Table 1), which displayed IC_50_ value below 100 nM in the DNA supercoiling assay, were tested against the human TopoIIα in DNA relaxation assay and against the human Hsp90α NTD in fluorescence thermal shift assay. Compounds **1** and **2** were identified as active compounds in the screening against GyrB pharmacophore models and as inactive compounds in the activity profiling against human TopoII and Hsp90 pharmacophore models (Figure 15). The substituted pyrrolamide moiety of compounds **1** and **2** matched the HBA and HBD features associated with Asp81 and the structured water molecule, while chlorine atoms matched the hydrophobic features. The benzothiazole and 4,5,6,7-tetrahydrobenzothiazole moieties of **1** and **2**, respectively, matched the aromatic ring feature associated with Arg84, while the carboxylate groups of **1** and **2** aligned well with the HBA and positive ionizable features associated with Arg144. Novobiocin, which was used as a positive control, was not retrieved by any of the models. Biological evaluation of **1** and **2** for binding to Hsp90α NTD and inhibition of TopoII confirmed results of the in silico selectivity prediction as they were found to be devoid of activity in concentrations up to 100 μM (Table 1).

Similarly, our potent Hsp90 NTD inhibitors **3** and **4** identified only by the **LB-Hsp90-Model-6**, with K_d_ values in the low nanomolar range were shown to be inactive against TopoII and *E. coli* DNA gyrase with IC_50_ values greater than 100 μM (Table 1) (Figure 15). The substituted resorcinol moiety of **5** and **6** corresponded to the complex network of HBA, HBD, aromatic and hydrophobic features, while additional aromatic and HBA features were aligned with the thiadiazole and phenyl moieties. Moreover, TopoII inhibitors **5** and **6** with low micromolar potency, were shown to be selective over Hsp90α NTD and DNA gyrase (Table 1) as also supported by pharmacophore model-based activity profiling (Figure 15). The complex pattern of the aromatic ring feature surrounded by multiple HBA features was matched by the central substituted 1,3,5-triazin-2(1H)-one scaffold, while other aromatic rings and hydrophobic features were aligned with the benzyl groups at positions 4 and 6. Etoposide does not bind to the TopoII ATP-binding site and was not a hit in activity profiling using TopoII models or the other models. Moreover, compound **7** developed as a selective nanomolar inhibitor of human carbonic anhydrase IX isoform was shown to be an inactive compound in both in silico (Figure 15) and in vitro profiling experiments (Table 1).

### 2.4. Molecular Docking

**Figure 16:**
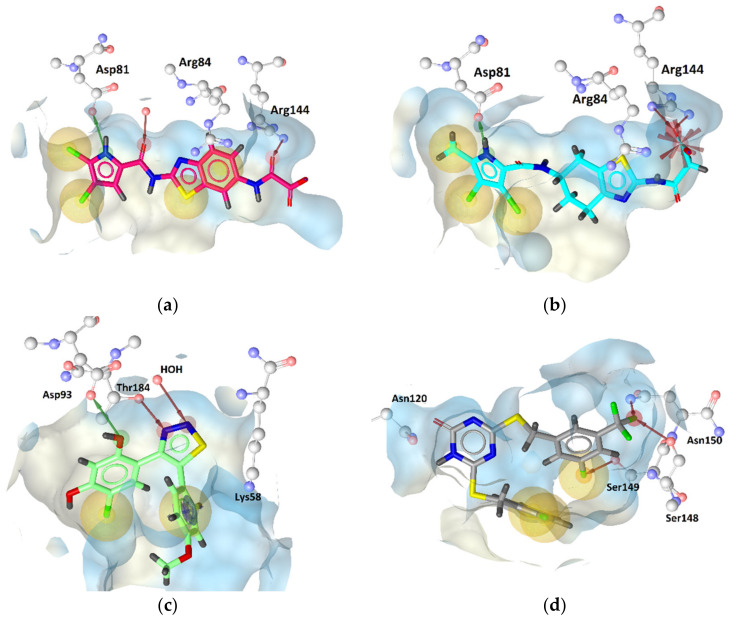
Docking binding modes of (**a**) compound **1**, (**b**) compound **2** in the GyrB, (**c**) compound **3** in the Hsp90, and (**d**) compound **5** in the TopoII ATP-binding sites.

Molecular docking experiments were performed to predict the binding modes of compounds **1**–**6** (Table 1) at the ATP-binding sites of GyrB, Hsp90, and TopoII. The docking binding modes of compounds **1** and **2** (Figure 16a,b) were similar to those of the co-crystallized pyrrolamide inhibitor (Figure 3a). Both inhibitors formed hydrogen bonds with Asp81 and additional hydrophobic interactions with chlorine atoms on the pyrrole ring. In addition, important hydrogen bonds and/or salt bridges were formed with the Arg144 side chain, a residue important for achieving selectivity for bacterial DNA gyrase. The predicted binding modes correlated well with the orientations of the compounds in pharmacophore models. In addition, compounds **3** (Figure 16c) and **4**, which formed hydrogen bonds with Asp93, Thr184, and a cation-π interaction with Lys58, were predicted to have similar binding modes to the co-crystallized Hsp90 inhibitor. The least reliable was the prediction of the binding mode of compounds **5** and **6** at the TopoII binding site as no crystal structure of TopoII in complex with the catalytic inhibitor has yet been solved. Nevertheless, the predicted binding mode of compound **5** at the TopoII active site (Figure 16d) was consistent with that reported previously [52]. The 1,3,5-triazin-2(1*H*)-one core was located near Asn120 with the potential to form hydrogen bonds, while other hydrophobic interactions and hydrogen bonds were predicted for benzyl substituents at positions 4 and 6 of the central scaffold.

## 3. Materials and Methods

### 3.1. Enzymes and Chemical Compounds

Production and purification of recombinant Hsp90α NTD have been described previously [54]. The molecular weight of the protein has been confirmed by high-resolution mass spectrometry, purity by SDS-PAGE, and the concentration was determined by UV-VIS spectrophotometry at 280 nm. The synthesis, chemical structure determination, and purity of compounds **1-7** have been previously described [4,48,49,50,51,52,53].

### 3.2. Software

The generation and validation of 3D structure- and ligand-based pharmacophore models were performed using LigandScout 4.4 Expert, available by Inte:Ligand GmbH (Vienna, Austria) [29,55]. Multi-conformational compound libraries were created with LigandScout’s conformer generator i:Con [56,57]. Activity profiling was performed in InteLigand’s activity profiling algorithm as implemented into a KNIME extension and used in KNIME Analytics Platform [58] using LigandScout Expert KNIME Extensions [59], which enables virtual screening of the multiconformational compound libraries against a set of 3D-pharmacophore models in parallel and generation of a heat map showing a profile of each ligand.

**Figure 17:**
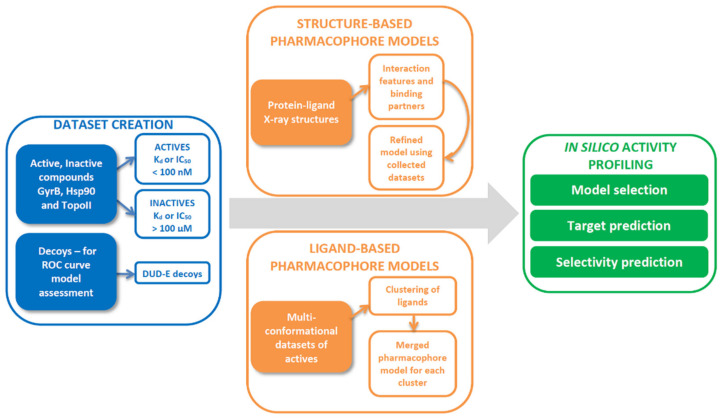
Dataset creation and 3D-pharmacophore modeling workflow used to identify best performing models for in silico target prediction and selectivity profiling of chemical structures.

A schematic representation of the workflow for the development of 3D-pharmacophore models is presented in Figure 17.

### 3.3. Identification of Proteins with Similar ATP-Binding Pockets

Human proteins with similar binding sites to bacterial GyrB were identified by ProBiS, a Web server for comparison of protein binding sites based on local structural alignments [37,38].

### 3.4. Compound Library Preparation

The data for active and inactive compounds against all studied enzymes were extracted from the ChEMBL database in November 2019. The reliability of the data in ChEMBL was checked with the original publications. In addition to true inactive compounds from the ChEMBL database, a set of decoys was generated for the most potent compounds against each target enzyme using the DUDE decoys database [43]. Decoys are theoretical structures, which have not been tested experimentally at the respective targets and were originally developed for testing docking-based virtual screening results. The decoys were used in this study for the generation of receiver operating characteristics (ROC) curves to compare and assess the performance of the generated pharmacophore models. A subset of the most potent compounds for each target was submitted to the DUDE decoy online generator, which resulted in 50 decoys per compound with similar 1D physicochemical properties but dissimilar 2D topology in comparison to the active compounds.

For calculation of active, inactive, and decoy compounds multi-conformational libraries, conformations were generated using i:Con with the default “FAST” settings [Timeout (s): 600, RMS threshold: 0.5, energy window: 15.0, max. pool size: 4000, max. fragment build time: 30, max. number of conformers: 25] and the virtual screening libraries were calculated using LigandScout’s idbgen algorithm.

### 3.5. Structure-Based Pharmacophore Modelling

For structure-based pharmacophore model generation in LigandScout, crystal structures of GyrB (PDB IDs: 3TTZ, 4P8O), Hsp90α (PDB IDs: 3TUH, 4U93) in complex with inhibitors were retrieved from the Protein Data Bank and interaction features with binding site amino acids were derived automatically using the direct approach and default settings. In addition, an ensemble of exclusion volume spheres was generated automatically, which represents restricted areas based on the enzyme binding site interaction partners. As in the case of topoisomerase IIα, there is no crystal structure of the enzyme in complex with the ATP-binding site inhibitor available, no structure-based pharmacophore model for topoisomerase IIα could be computed.

### 3.6. Ligand-Based Pharmacophore Modeling

All ligands in the actives datasets (221 GyrB inhibitors, 649 Hsp90 inhibitors, 21 TopoII inhibitors) were clustered in LigandScout using pharmacophore RDF-code similarity or pharmacophore alignment score with default settings. For each cluster of inhibitors with more than five members, ligand-based pharmacophore models were generated using the merged feature pharmacophore approach using the default settings (number of omitted features for merged pharmacophore: 4, partially matching features optional, threshold (%): 10.0). The creation of exclusion volume spheres around the alignment of the ligands was also enabled. For each ligand-based pharmacophore model, ligands of the cluster, used to generate the model, were set as the training set. All ligands of the training set were automatically aligned to the generated pharmacophore model.

### 3.7. Pharmacophore Model Optimization

Initial structure- and ligand-based pharmacophore models were used to screen a dataset of active compounds and a dataset of decoy and inactive molecules with a scoring function set to “pharmacophore-fit” and the screening mode set to “match all query features” with a maximum number of omitted features set to zero. The performance of all individual models were assessed by ROC curve analysis. In most cases, structure-based pharmacophore models were too restrictive and identified only a small number of true actives from the dataset. In the next steps, selected features were omitted from the pharmacophore models or selected HBA or HBD vector features were converted to non-directional HBA or HBD features, or the tolerance of a selected feature was increased or selected feature was marked optional. All these possible modifications and their combinations were considered to increase the number of true actives while keeping the number of inactive compounds as low as possible.

### 3.8. Molecular Docking

Compounds **1** and **2** were docked to the GyrB (3TTZ.pdb), compounds **3** and **4** to the Hsp90 (3TUH.pdb) and compounds **5** and **6** to the TopoII (4R1F.pdb) ATP-binding sites using AutoDock Vina 1.1 [60] as built-in in LigandScout 4.4. The following default settings were used for molecular docking: Exhaustiveness: 8, Max. number of modes: 9, Max. energy difference: 3.

### 3.9. Determination of Inhibitory Activities on E. coli DNA Gyrase

The DNA supercoiling assay from Inspiralis for the determination of IC_50_ values was performed according to previously reported procedures [2].

### 3.10. Determination of Inhibitory Activities on Human DNA Topoisomerase IIα

Inhibitory activities were determined in an assay from Inspiralis on streptavidin-coated 96-well microtitre plates from Thermo Scientific Pierce. First, the plates were rehydrated with buffer (20 mM Tris·HCl, 0.01 % *w*/*v* BSA, 0.05 % *v*/*v* Tween 20, 137 mM NaCl, pH 7.6) and then biotinylated oligonucleotide was immobilized. After washing off the unbound oligonucleotide, the enzyme assay was performed. The reaction volume of 30 μL in buffer (50 mM Tris·HCl, 10 mM MgCl_2_, 125 mM NaCl, 5 mM DTT, 0.1 μg/mL albumin, 1 mM ATP, pH 7.5) contained 1.5 U of human DNA topoisomerase II, 0.75 μg of supercoiled pNO1 plasmid, and 3 μL of an inhibitor solution in 10% DMSO containing 0.008% Tween 20. Reaction solutions were incubated at 37 °C for 30 min. After that, the TF buffer (50 mM NaOAc, 50 mM NaCl, and 50 mM MgCl_2_, pH 5.0) was added to terminate the enzymatic reaction. After additional incubation for 30 min at RT, during which biotin–oligonucleotide–plasmid triplex was formed, the unbound plasmid was washed off using TF buffer, and Diamond Dye in T10 buffer (10 mM Tris·HCl, 1 mM EDTA, pH 8.0) was added. The fluorescence was measured with a microplate reader (BioTek Synergy H4, excitation: 485 nm, emission: 537 nm). Initial screening was done at 100 or 10 μM concentrations of inhibitors. For the most active inhibitors, IC_50_ was determined using seven concentrations of tested compounds. GraphPad Prism 6 software was used to calculate the IC_50_ values. The results are reported as the average value of three independent measurements. As a positive control, etoposide (IC_50_ = 71 μM) was used.

### 3.11. Determination of Binding to Hsp90

Compound binding to Hsp90 was determined by the fluorescence-based thermal shift assay (FTSA) which determines the thermal stability of the free and ligand-bound protein [61,62,63]. The stabilization efficiency is directly related to the ligand-binding affinity. The experiments were carried out with Corbett Rotor-Gene 6000 (QIAGEN Rotor-Gene Q) spectrofluorimeter (using excitation 365 ± 20 nm and detection at 460 ± 15 nm). Protein solutions in the presence of various concentrations of a compound were heated from 25 to 95 °C at a rate of 1 °C/min. Protein unfolding was monitored by measuring the fluorescence of 8-anilino-1-naphthalenesulfonate (ANS) dye at added 50 μM concentration. Increasing concentration of a compound shifted the protein denaturation temperature increasingly upwards depending on the binding affinity. Samples contained 5 μM protein, ligand concentration ranging from 0 to 200 μM, 50 μM sodium phosphate buffer (pH 7.5) containing 100 μM NaCl and 2% DMSO. Protein melting temperatures were determined by fitting the fluorescence curves while the compound dosing curves were simulated as described previously. All experiments were repeated at least twice.

## 4. Conclusions

We developed predictive and selective on-target GyrB and off-target TopoIIα and Hsp90 3D-chemical feature-based pharmacophore models using LigandScout. Structure-based pharmacophore models were created based on X-ray derived enzyme-inhibitor complexes, while ligand-based models were created based on the known potent ligands. The models were validated and trained using sets of known active, inactive, and decoy molecules. The models identified common interaction features in the three ATP binding sites and also unique features associated with selective binding. The activity profiling of our GyrB inhibitors using these off-target pharmacophore models predicted their selective on-target binding. These results were supported also by in vitro assays on Hsp90 and TopoIIα for selected compounds **1** and **2**. Moreover, selective Hsp90α NTD inhibition was predicted for **3** and **4**, which was also confirmed in in vitro assays against *E. coli* DNA gyrase and human TopoII. Likewise, selective TopoIIα inhibition was predicted and experimentally confirmed for compounds **5** and **6**. Our developed 3D-chemical feature-based pharmacophore models are therefore valuable tools for the prediction of activity and selectivity of known and novel GyrB inhibitors.

## Figures and Tables

**Table 1:** Experimental in vitro activity profiling of DNA gyrase inhibitors **1** and **2**, Hsp90α NTD inhibitors **3** and **4**, TopoIIα inhibitors **5** and **6**, and carbonic anhydrase IX inhibitor **7**.

Compound	Structure	Human Hsp90α NTD K_d_ [μM]	Human TopoII IC_50_ [μM]	*E. coli* DNA gyrase IC_50_ [μM]
ICPD47		0.0125 [49,50]	/	/
novobiocin		/	/	0.17
etoposide		/	71	/
**1**	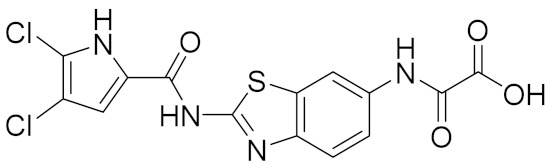	>100	>100	0.087 [4]
**2**	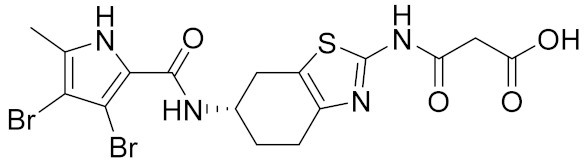	>100	>100	0.020 [48]
**3**	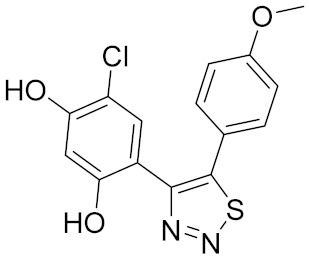	0.026 [50]	>100	>100
**4**	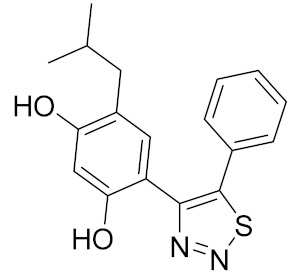	0.0066 [51]	>100	>100
**5**	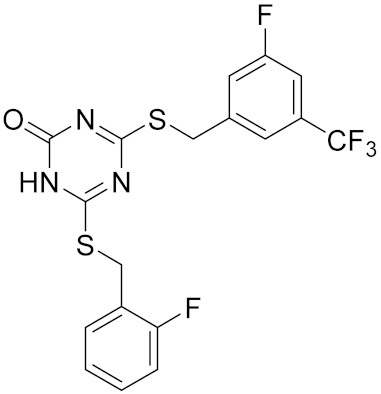	>100	8.1 [52]	>100
**6**	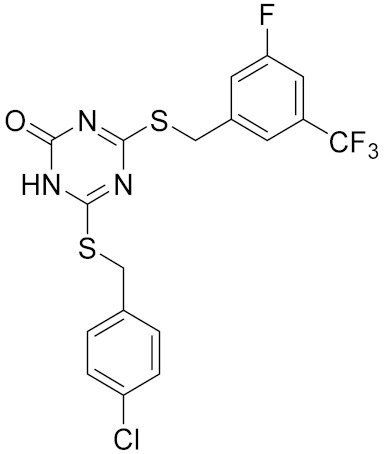	>100	8.4 [52]	>100
**7** [53]	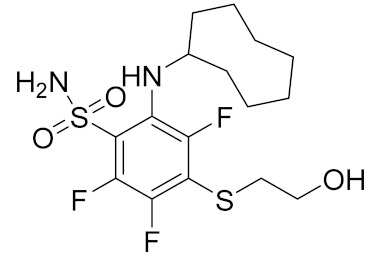	>100	>100	>100

## Data Availability

Data is contained within the article and Supplementary Materials.

## Supplementary Materials

The following are available online at https://www.mdpi.com/article/10.3390/ph14080789/s1, Figure S1: Validation phase of (**a**) **LB-GyrB-Model-5m**; (**b**) **LB-GyrB-Model-4**; (**c**) **LB-GyrB-Model-6** ligand-based pharmacophore models; Figure S2: Validation phase of (**a**) **LB-Hsp90-Model-3**, (**b**) **LB-Hsp90-Model-4**, (**c**) **LB-Hsp90-Model-5**, and (**d**) **LB-Hsp90-Model-6** Hsp90 ligand-based pharmacophore models; **Figure S3.** Validation phase of (**a**) **LB-TopoII-Model-1**, (**b**) **LB-TopoII-Model-2**, (**c**) **LB-TopoII-Model-3**, and (**d**) **LB-TopoII-Model-4** TopoII ligand-based pharmacophore models.
