# Supplementary material for: Selective DNA Gyrase Inhibitors: Multi-Target in Silico Profiling with 3D-Pharmacophores
Source: Pharmaceuticals (Basel). 2021 Aug 10;14(8):789. doi: 10.3390/ph14080789 (PMC8400042; doi:10.3390/ph14080789)
Supplement: Supplementary file 1 [file pharmaceuticals-14-00789-s001.zip › pharmaceuticals-1305006 - revised - tracked changes.pdf]

*Supplementary Material*

# Selective DNA Gyrase Inhibitors: Multi-Target *In Silico* Profiling with 3D-Pharmacophores

**Tihomir Tomašič [1,*], Asta Zubrienė [2], Žiga Skok [1], Riccardo Martini [3,4], Stane Pajk [1], Izidor Sosič [1], Janez Ilaš [1], Daumantas Matulis [2], and Sharon D. Bryant [3]**

1 University of Ljubljana, Faculty of Pharmacy, Aškerčeva 7, Ljubljana, Slovenia; ziga.skok@ffa.uni-lj.si; stane.pajk@ffa.uni-lj.si; izidor.sosic@ffa.uni-lj.si; janez.ilas@ffa.uni-lj.si
2 Vilnius university, Life Sciences Center, Institute of Biotechnology, Department of Biothermodynamics and drug design, Saulėtekio 7, Vilnius LT-10257, Lithuania; astzu@ibt.lt; matulis@ibt.lt
3 Inte:Ligand Softwareentwicklungs- und Consulting GmbH, Mariahilferstrasse 74B, Vienna, Austria; bryant@inteligand.com
4 Discngine S.A.S., 79 Avenue Ledru Rollin, 75012 Paris, France; riccardo.martini@discngine.com
* Correspondence: Tihomir.tomasic@ffa.uni-lj.si; Tel.: +386-1-4769-556

## 1. ROC plots from the GyrB ligand-based pharmacophore model validation.

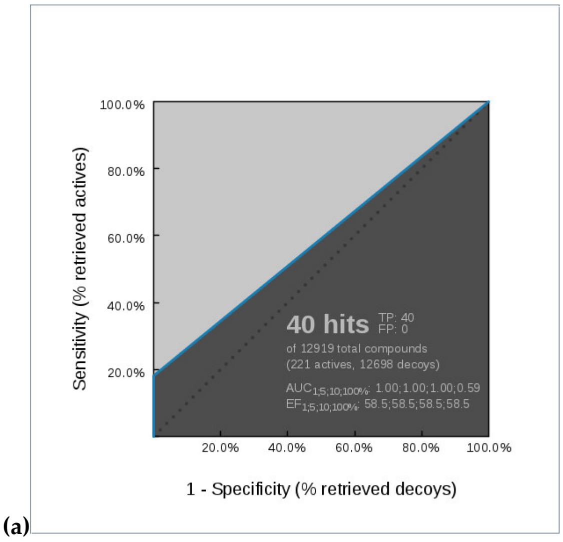


(a)

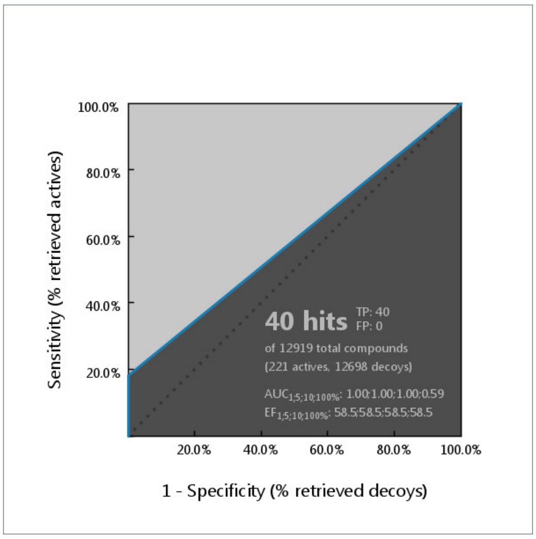


(b)

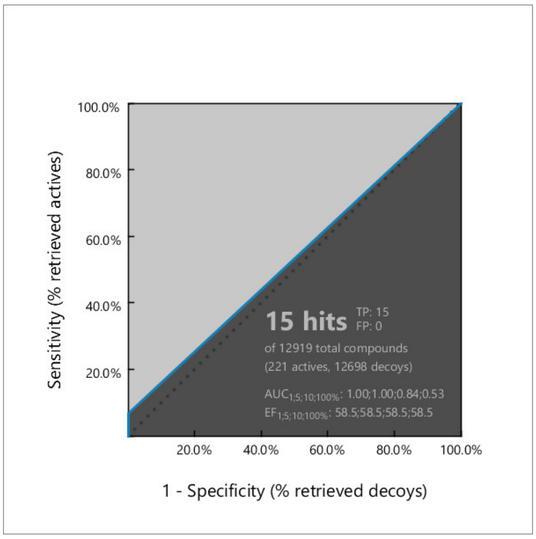


(c)

**Figure S1.** Validation phase of **(a) LB-GyrB-Model-5m**; **(b) LB-GyrB-Model-4**; **(c) LB-GyrB-Model-6** ligand-based pharmacophore models. Resulting ROC plot (curve shown in blue) from virtually screening 12919 compounds (221 GyrB actives and 12698 generated decoys) with the ligand-based pharmacophore model. TP = true positives; FP = false positives; AUC = area under the curve; EF = enrichment factor.

## 2. ROC plots from the Hsp90 ligand-based pharmacophore model validation.

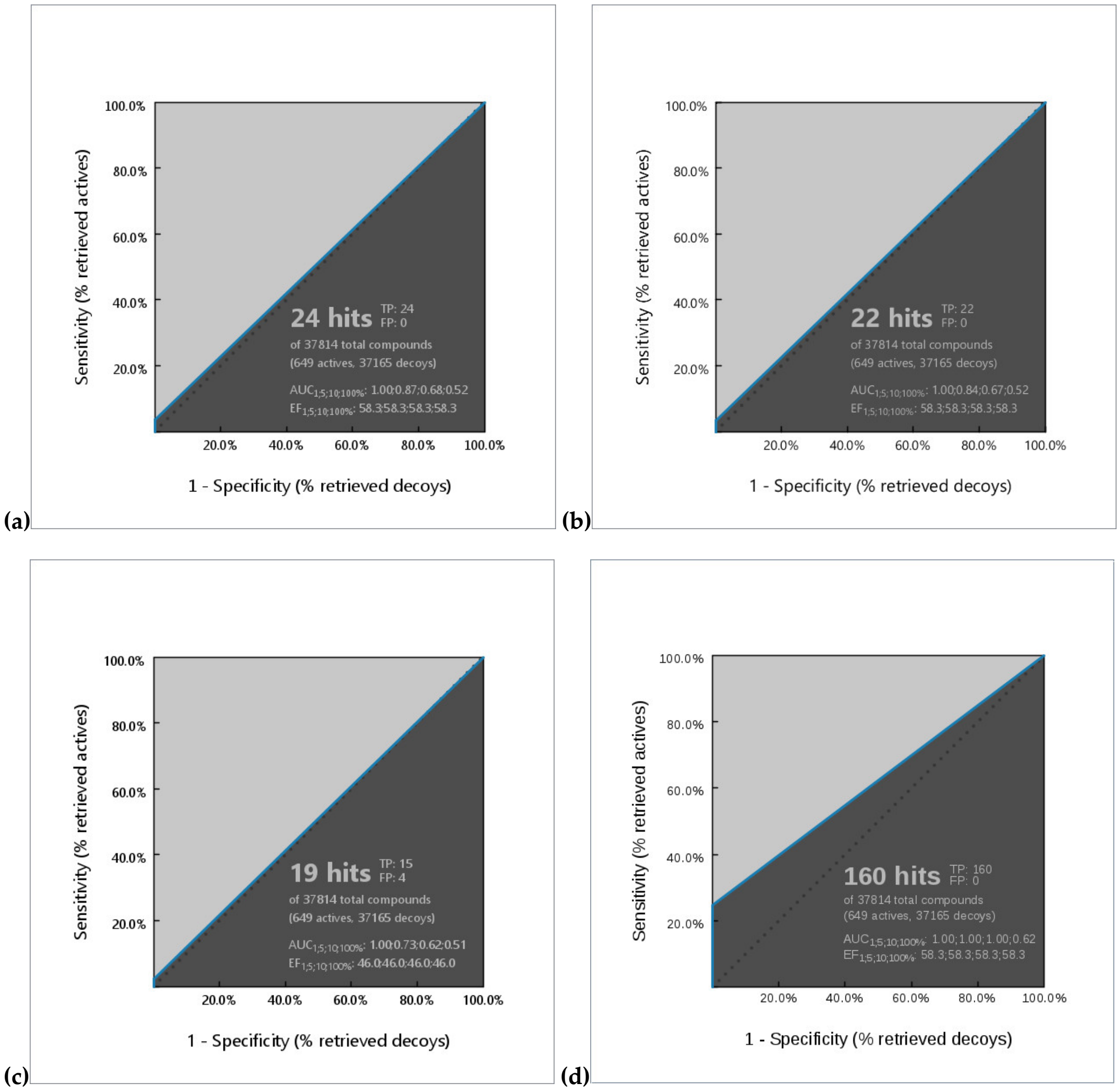


**Figure S2.** Validation phase of **(a) LB-Hsp90-Model-3**, **(b) LB-Hsp90-Model-4**, **(c) LB-Hsp90-Model-5**, and **(d) LB-Hsp90-Model-6** Hsp90 ligand-based pharmacophore models. Resulting ROC plot (curve shown in blue) from virtually screening 37814 compounds (649 Hsp90 actives and 37165 generated decoys) with the ligand-based pharmacophore model. TP = true positives; FP = false positives; AUC = area under the curve; EF = enrichment factor.

### 3. ROC plots from the TopoII ligand-based pharmacophore model validation.

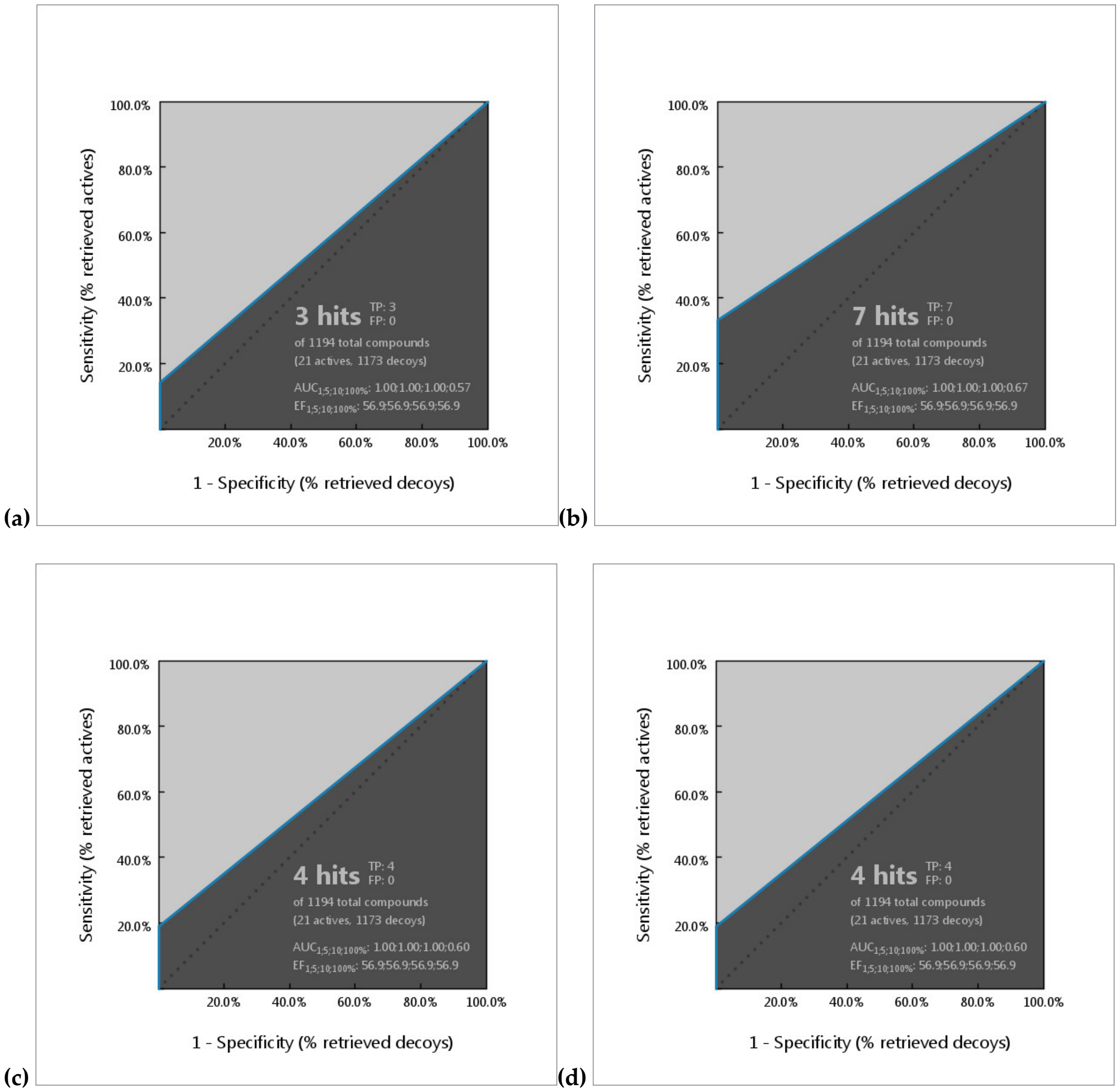


**Figure S3.** Validation phase of **(a) LB-TopoII-Model-1**, **(b) LB-TopoII-Model-2**, **(c) LB-TopoII-Model-3**, and **(d) LB-TopoII-Model-4** TopoII ligand-based pharmacophore models. Resulting ROC plot (curve shown in blue) from virtually screening 1194 compounds (21 TopoII actives and 1173 generated decoys) with the ligand-based pharmacophore model. TP = true positives; FP = false positives; AUC = area under the curve; EF = enrichment factor.
